# Non-Binary Clients’ Experiences of Psychotherapy: Uncomfortable and Affirmative Approaches

**DOI:** 10.3390/ijerph192215339

**Published:** 2022-11-20

**Authors:** Fau Rosati, Maric Martin Lorusso, Jessica Pistella, Guido Giovanardi, Bianca Di Giannantonio, Marta Mirabella, Riccardo Williams, Vittorio Lingiardi, Roberto Baiocco

**Affiliations:** 1 Department of Developmental and Social Psychology, Faculty of Medicine and Psychology, Sapienza University of Rome, 00185 Rome, Italy; 2 Department of Psychology, Campus Cesena, Alma Mater Studiorum, University of Bologna, 47521 Bologna, Italy; 3 Department of Dynamic and Clinical Psychology, and Health Studies, Faculty of Medicine and Psychology, Sapienza University of Rome, 00185 Rome, Italy

**Keywords:** non-binary, gender identity, psychotherapy, psychotherapists, affirmative approach, microaggression, microaffirmation, therapeutic relationship, misgendering, cisgenderism

## Abstract

Non-binary people may face specific challenges in psychological settings. Psychotherapists often display a lack of preparation for non-binary gender identities, resulting in overt or subtle forms of prejudice that compromise the therapeutic alliance. The present study aimed to provide data on non-binary clients’ positive and negative experiences within therapeutic relationships. Twenty-five interviews were conducted with non-binary people in the age range of 19–35. Using codebook thematic analysis, the researchers identified three main themes: (1) the self of the psychotherapist, consisting of the impact of the therapist’s personal (i.e., sexual identity) and professional (i.e., competence) characteristics on the therapeutic experience; (2) the practice of the psychotherapist, emerging as affirmative (validation and microaffirmations) or negative (gender identity change efforts, manifest aggressions, and microaggressions) approaches toward non-binary identities; (3) the therapeutic relationship, referring to the alliance, rupture, and reparation based on the therapists’ openness toward non-binary identities. To provide a safer setting for non-binary clients, psychotherapists should incorporate issues related to gender minority identities in their training, acknowledge clinical errors when they occur and adopt an active predisposition to learn through the client’s experience, giving value to their unique contribution.

## 1. Introduction

Non-binary is an umbrella term to refer to those people who perceive their gender as falling outside the binary construct of “man” or “woman”. There is evidence that non-binary people are less likely to access competent psychological care than binary transgender (or trans) and cisgender (or cis) people. Non-binary people may face specific challenges when dealing with healthcare providers, such as psychotherapists, who often display a lack of preparation resulting in overt or subtle forms of prejudice [1]. The present study aimed to fill this gap by enriching the psychological literature about the experiences of non-binary people in psychotherapy contexts.

The experience of gender among non-binary individuals varies and may include identifying with both male/female genders (e.g., bigender), a third gender, no gender (e.g., agender), or moving between binary genders (e.g., genderfluid). Conceptually, non-binary identities fall under the transgender umbrella since their gender differs (at least partly) from their sex assigned at birth. However, not all non-binary people consider themselves transgender, as the clear distinction between trans and cis identities can be perceived as an additional limiting dichotomy [2].

Historically, Western societies’ psychological and scientific communities have denied, denigrated, and pathologized all people that do not align with their assigned gender at birth, incorporating a systemic ideology named “cisgenderism” [3]. Cisgenderist (or cisnormative) ideology considers cis and binary identities normal, healthier, and superior to trans and non-binary identities, reinforcing non-binary negativity and increasing health disparities between the cis/binary and the trans/non-binary populations [4]. Recent studies have shown that non-binary people have worse mental health outcomes when compared with both cis [5,6] and binary trans people [7,8,9]. Such worse mental health conditions may be explained by considering the unique forms of stigma affecting the non-binary population. Indeed, non-binary people face challenges in being recognized as having valid gender identities and experience a high rate of social erasure and invisibility [2]. 

The non-binary erasure reflects the absence of alternatives other than the male/female options in official documents, public bathrooms, and locker rooms, as well as within broader social beliefs, which expose non-binary people to the risk of being non-affirmed by others and/or in the condition of educating them, thus receiving less support from significant others and health and social care providers [10]. There is evidence that non-binary people are less likely to access gender-affirming care [11,12] compared to binary trans people, and that they face specific challenges when dealing with healthcare providers, such as psychotherapists, who often use a binary perspective and gendered language [13,14]. 

The lack of preparation of psychotherapists results in overt or subtle forms of prejudice concerning non-binary people, such as non-binary negativity, microaggressions, and unfamiliarity with non-binary issues [15,16,17]. Negative practices toward non-binary people in psychotherapy are motivated by non-acceptance and may manifest as aggressive and unprofessional practices such as negatively judging, pathologizing, and not recognizing non-binary genders [18]. Microaggressions are more subtle forms of verbal and non-verbal discrimination toward a marginalized social group [19,20]. They are often exerted unconsciously and expressed through apparently insignificant or harmless practices, making them difficult to identify [21]. As the opposite of microaggressions, microaffirmations are small interpersonal practices or gestures that communicate validation for an identity [22]. The psychotherapist can actively validate non-binary clients’ experiences and affirm their gender identity through empowering interactions [23,24]. 

A growing number of people, especially among youth, self-identify as non-binary [25,26]. The study was conducted in Italy, where non-binary people and issues are invisibilized and underrepresented [27]. The legislation in Italy does not allow a third legal and administrative option for genders that fall outside the male/female dichotomy. Moreover, Italian is a strongly gendered language: the gender (male or female) of the person must always be immediately perceivable, using pronouns, suffixes, and gendered articles. The risk of misgendering in verbal interactions is, therefore, very high, especially if compared to other languages that do not share the same gendered linguistic structure. Feminist linguistic claims have contributed to deconstructing the fictitious neutrality of using plural male-gendered language when referring to a group of people [28]. In recent times, transfeminist and queer claims have increasingly fostered the use of a gender-neutral language, for instance, suggesting the use of “schwa” (ə) as a neutral suffix in both written and spoken language. This latter linguistic solution might represent a good strategy to address and refer to non-binary people [29].

Due to social stigma and erasure, such a population presents elevated adverse mental health outcomes and may need increased access to psychotherapy. Previous studies involving gender minority individuals documented that negative experiences occurring in mental health settings damage the therapeutic alliance and, consequently, the therapeutic outcomes [19,30,31]. There is a lack of research conducted on non-binary clients, and little is known about what makes a positive or negative psychotherapeutic experience and how these people choose a psychotherapist. The present study aimed to fill this gap by enriching the psychological literature about the experiences of non-binary people in psychotherapy. To understand how to create a safer and positive setting for non-binary clients and effectively meet their needs, it is essential to enhance awareness about those aspects that make a psychotherapeutic experience affirmative and healing.

## 2. Materials and Methods

### 2.1. Procedure and Positionality

The different steps of the research (i.e., study design, interview creation, participants’ recruitment, data collection, and data analysis) were guided by the authors, who are researchers and psychologists directly engaged in the clinical work with non-binary clients. The research members embodied different positionalities concerning their academic role, age, gender identity, and sexual orientation (e.g., non-binary, cisgender, queer, lesbian, gay, heterosexual, full professors, researchers, psychologists, and/or interns). All the interviews were conducted by trained students and researchers, some of whom had a non-binary gender identity and/or a queer/lesbian sexual orientation.

The interviewers were open about their gender identity and sexual orientation to let participants feel free to choose with whom to interact during the interview. Therefore, the interview setting was perceived as less at risk of cisnormative practices, thus enhancing participants’ motivation and openness to participate in the study. During the recruitment phase, participants were informed about the general content and the average time required for the interview (30 min). Before starting the data collection, they were asked to sign their consent to proceed. The study was conducted according to the guidelines of the Declaration of Helsinki and approved by the Ethics Committee of the Department of Developmental and Social l Psychology, Sapienza University of Rome (protocol number: 0001088; date of approval: 9 September 2021).

### 2.2. Instruments for Data Collection

Data were collected using a brief questionnaire for demographic and gender identity-related information, followed by a semi-structured interview. Demographic information included age, nationality, sex assigned at birth (with the option of not declaring it), education level, socioeconomic status, employment status, and living and relationship conditions. The Gender Diversity Questionnaire (GDQ) [25,32] was used to obtain specific information about participants’ use of pronouns, open descriptions of their gender identity, identification with the transgender category, access to medical treatment (e.g., hormones, surgery), coming out, and perception of their gender in social interactions. 

The interview protocol was developed ad hoc by the research team. Given the lack of previous research on non-binary clients’ psychotherapy experiences, questions were designed based on the research team’s personal and professional experiences, as well as on the existing literature on broader Lesbian, Gay, Bisexual, Trans, Queer, Intersex, and Asexual (LGBTQIA+) clients’ psychotherapy experiences. The interview protocol allowed obtaining in-depth information relating to the following thematic areas: (a) the centrality of gender identity in psychotherapy (e.g., “How central have been the theme of gender identity in your personal therapy compared to other issues of your life?”); (b) the impact of the psychotherapist’s identity (e.g., sexual orientation/gender identity, knowledge of non-binary issues, engagement in trans and non-binary communities) on the client’s choice and evolution of therapy (e.g., “Do you know your therapist’s gender identity/sexual orientation?”; “What are the positive and negative aspects of having a [cis/trans/non-binary/heterosexual/non-heterosexual] therapist?”); (c) the psychotherapist’s attitude towards non-binary gender identities (e.g., “What kind of attitude do you think your therapist has towards non-binary identities?”); (d) the psychotherapist’s use of the client’s chosen name and pronouns (e.g., “Did you feel that your therapist respected and valued your chosen name and pronouns?”); (e) the psychotherapist’s reactions toward the client’s coming out (e.g., “What was your therapist’s reaction when you came out as non-binary? Do you think they correctly explored the dynamics associated with your coming out in the different contexts of life?”); (f) episodes of alliance, ruptures and potential repair (e.g., “Can you tell an episode where your therapist disappointed you or showed a lack of sensitivity towards you as a non-binary person?”; “If yes, has your therapist noticed this? Did they pay attention to what had happened in psychotherapy trying to regain the lost harmony?”). The interview protocol will be made available upon request to the corresponding author.

### 2.3. Participants

The inclusion criteria for this study were to be a non-binary person attending or having attended psychotherapy within the last five years. Recruitment was carried out through the convenience sampling technique, spreading an online announcement on social media, as well as through personal acquaintances and connections of the research team. A total of 25 non-binary people in the age range 19–35 (*M* = 27.44; *SD* = 4.31) participated in the study. All participants were White and Italian. Most of them (n = 21) were assigned female at birth. Some participants used additional categories to describe their gender identity: “genderqueer” (n = 2), “genderfluid” (n = 3), “agender” (n = 3), “bigender” (n = 1), and “demigirl” (n = 1) [32]. Most participants (n = 15) used more than one pronoun (e.g., both he/she or a mix that included neutral pronouns). Some participants used neutral pronouns (n = 3), while the rest used he/him (n = 5) and she/her (n = 2). Since Italian is a strongly gendered language that does not provide for recognized neutral pronouns and suffixes, Italian non-binary people commonly switch between the standard pronouns he/she. 

Interestingly and coherently with previous studies, only half of the participants (n = 13) self-identify as trans [33]. This data may depend on different reasons, such as the transnormative dominant discourse that legitimizes only the binary and medical trans experiences, thus leading non-binary people—especially those who do not opt for a medical transition—to feel they are not trans enough [34,35]. Alternatively, given that gender perception significantly varies among non-binary people, someone may perceive the “trans” category as excessively rigid and unrepresentative of their experience [2]. Finally, only a few participants accessed gender-affirming medical treatment (n = 3): This can be due to the additional challenges that non-binary people face when they try accessing gender-affirming care [11,12], as well as to a less perceived need to transform one’s body [36].

Concerning sexual orientation, participants described themselves as lesbian (n = 4), gay (n = 1), bisexual (n = 8), pansexual (n = 3), queer (n = 4), and unlabeled (n = 5). Regarding additional demographic information, the majority of participants had an average (n = 20), followed by low (n = 3) and high (n = 2) socioeconomic status. Their educational level varied from high school diploma (n = 8), bachelor’s degree (n = 14), and doctoral degree (n = 3).

### 2.4. Analytical Approach

The interviews’ transcripts were analyzed using Codebook Thematic Analysis, specifically Template Analysis [37]. Codebook Thematic Analysis combined the coding reliability approach with the values of reflexivity Thematic Analysis, understanding researcher subjectivity as a resource for research, and data coding and interpretation as an inherently and unavoidably subjective practice [38]. The analysis followed different steps. In the first, the first and second authors independently generated a series of codes using a selection of five common transcripts. A code is typically a word or short phrase that symbolically assigns a summative, salient, essence-capturing, or attribute to a portion of data [39]. Authors put in the fieldwork both their embodied positionality as non-binary subjectivities who have experienced psychotherapy as clients and their knowledge as professionals in the psychological fieldwork. Moreover, the coding phase was informed by the model of microaggression and microaffirmation [23]. In the second step, the same authors discussed and redefined the initial codes, which were used as a template to create a common codebook to analyze all the transcripts. Themes were generated as topic summaries in a third step, where the other research team members were involved in the (re)definition of the themes and sub-themes to conceptualize the final structure. The other authors, all cisgenders, highlighted how, in the coding phase, the first authors had underestimated the presence of microaggressions. The team discussed together how the non-binary positionality of the authors could have made them afraid to be perceived as biased, thus underestimating the phenomenon. The team recognized this mechanism as a source of internalized stigma of non-binary authors. In a fourth step, the two authors came back to the transcripts and renamed, where necessary, each extract’s category within the final structure. Finally, the two authors divided in half the participants’ group in order to independently re-code the extracts of all participants, using the final thematic structure.

## 3. Results

Most of the participants (n = 15) did not know the sexual orientation of their therapist; some of them declared that their therapist was heterosexual (n = 6), lesbian (n = 3), and gay (n = 1). Nine participants did not know or wanted to assume the gender identity of their therapist, while the rest reported that their therapist was a cisgender woman (n = 11), a cisgender man (n = 4), or non-binary (n = 1). Finally, most of the respondents (n = 17) considered it important to know their therapist’s gender identity and sexual orientation.

Through codebook thematic analysis, we identified three main themes/topics that represent some key aspects of psychotherapy: (1) the self of the psychotherapist, that is, how the psychotherapist’s sexual identity and knowledge/experiences of non-binary issues impact the therapeutic experience; (2) the practice of the psychotherapist, which can be characterized by an affirmative (validation and microaffirmations) or a negative (gender identity change efforts, manifest aggressions, and microaggressions) approach toward non-binary identities; (3) the therapeutic relationship, consisting in the creation of the alliance, potential rupture, and reparation based on the perception that participants have of the psychotherapist’s openness toward their gender identity. The three themes and the related sub-themes must be considered as interconnected as, for instance, a lack of knowledge on non-binary issues may lead to a negative approach that, in turn, will cause a rupture of the therapeutic alliance. Table 1 reports each sub-theme’s thematic structure, representative quotations, and frequencies. 

### 3.1. The Self of the Psychotherapist

#### 3.1.1. Psychotherapist’s Gender Identity and Sexual Orientation

For some participants, the gender identity and, to a lesser extent, the sexual orientation of the therapist emerged as important factors that influenced the client’s choice of therapist. Someone expressed the desire to have a non-binary psychotherapist, expecting to be more at ease and comfortable in such a setting and assuming that non-binary therapists would likely be more competent thanks to the experiences related to their gender identity:

I’ve chosen a non-binary psychologist, for me it’s fundamental. This is the reason why I did not start and carried out psychotherapy until recently. Because a large part of my individuality and a large amount of my problems […] are filtered from my gender identity, and if the person in front of me is uninformed or unqualified, they will handle these problems in the wrong way and will not actually help me to deal with them.

(L., 23 years)

However, finding a non-binary therapist is extremely difficult in Italy, so the second choice fell on cis women that were preferred to cis men, regardless of sexual orientation:

I don’t care about sexual orientation. Regarding gender, I would like a non-binary therapist, but I don’t think there are many [laughs]. So, everything is fine as long as it is not a cis man because I feel he couldn’t fully understand. I believe that a woman can be more open to the question [of non-binary gender identity and sexuality], and, obviously, someone who is not cis, would understand it perfectly.

(M., 28 years)

Other participants did not consider the therapist’s sexual orientation and gender identity as factors impacting the therapist’s ability:

In my opinion, it is not important information, also because it is an extremely personal matter. I may be dealing with a male therapist who is cis but who still has a whole range of skills and openings.

(F., 28 years)

A few participants also reported negative experiences related to a non-binary therapist. Indeed, sharing a gender minority identity may have some benefits and stimulate unique resonances in both clients and therapists. In the case of clients, they may desire being validated and/or perceive being under evaluation:

Having a non-binary trans person in front of you can also be a problem in terms of being validated […] If I sit in front of you and you tell me “Ah, I’ve lived it too” I can say to myself “Okay, then I am” […] and if I start to think that it is you who must tell me how much trans or non-binary I am, you have a weapon in your hand that you have to know how to manage.

(A., 35 years)

Moreover, since the queer communities are often relatively small, non-binary psychotherapists and clients may share the same community and encounter relevant ethical dilemmas. One participant reported negative and confusing feelings due to unclear boundaries with their therapist:

My last therapist was queer and non-binary. She wasn’t professional because she took care of me even though we had a series of common relationships and political spaces. She didn’t put the right distances […] and so let’s say she has me… I felt a little manipulated because then I discovered things about her […] it was very traumatic as an end like I don’t know, for me it was helpful in its way, but it was not professional in my opinion.

(S., 26 years)

#### 3.1.2. Psychotherapist Knowledge of Non-Binary Issues

The therapist’s knowledge and competence in non-binary identities was a crucial topic in the participants’ narratives. Most of the therapists were described as not trained and/or unprepared, and several participants believed that it was quite impossible to find a therapist who was already prepared and competent on non-binary issues. As B. stated, “I think no one has good enough training on this stuff” (30 years). Others performed intensive research to ensure they did not find themselves in uncomfortable settings:

I made careful research as I thought that I could not go there to explain everything. And so, I found my therapist who deals with, let’s say, “queer issues” and I read a couple of papers she wrote, and I liked them, and so I said, “oh well, she seems to be the right person”.

(R., 31 years)

The issue of having to educate one’s therapist was central in participants’ narratives:

My therapist had never had anyone before who described themselves as I do. He is open-minded and ready to listen, but I had to explain a lot of things to him, sensations, emotions, what makes me uncomfortable, and so on. All these things must be explained in more detail since they don’t have an idea and there are no cultural references [of non-binarism].

(F., 28 years)

In some cases, participants reported having perceived that, for their therapist, having a non-binary client was an opportunity for training or “a particular case study”, as stated by F. (28 years). Such participants do not seem to be annoyed but, instead, they positively interpret the dynamic:

In my opinion, he did “mmm, very well, there is something for my studies” [laughs]. I think I’m an asset to him.

(V., 36 years)

This client’s positive interpretation was likely verified when the therapist’s lack of knowledge was associated with positive variables, such as open-mindedness and motivation to train themselves on non-binary issues:

I really appreciate her openness; she told me that during this year of therapy with me, she trained herself, both during the sessions with me and on her own, by studying and reading stuff to be able to understand and support me.

(R., 30 years)

However, the therapist’s openness to non-binary issues was insufficient to make a suitable intervention. As stated by one participant, the risk is that the therapist overemphasizes gender issues while remaining superficial on other meaningful aspects for the client:

It seems like he remains a bit superficial about the rest because he sees me as someone who has already faced many things. This likely depends on the fact that for him, it is incredible that someone could have gone through such an experience and understand a whole series of things on their own. On the other hand, I had the impression that he did not examine in depth some aspects of my life.

(L., 27 years)

Some participants reported having ended their therapy out of excessive frustration due to the idea of being “the expert” that trains the therapist on non-binary issues:

I’m in a bit of a crisis because I just left the therapist. After all, she didn’t have enough awareness of trans issues. I had to explain too many things, from my personal and intimate issues to social issues, right? Something that for me are totally connected, and she didn’t understand, and therefore I had to explain, and this made me angry.

(M., 26 years)

Finally, when participants encountered a therapist that was trained and engaged in trans issues/communities, their experience appeared empowered, and the therapeutic relationship could more easily benefit from trust:

She is trained, she is prepared, and she already knew non-binarism, gender fluidity, and so on. She told me that she had concrete experience with other trans patients […] I introduced myself to her, saying that something was happening to me in terms of identity, and the first things she said to me were about her experiences. She told me that she did her dissertation on trans issues, worked with trans women in prison and fought for them since they were put in jails with men. So, I said, “Well, top!”

(L., 24 years)

### 3.2. The Practice of the Psychotherapist

#### 3.2.1. Non-Binary Affirmative Approach through Validation and Microaffirmations

The therapist’s practice was characterized by verbal and non-verbal communication that strongly influenced the participants’ sense of comfort and safety. Participants’ description of the affirmative approach included the therapist’s act of seeing their authentic gender:

I’ve always had anxiety associated with the idea of starting the [medical] path because I think, “If I don’t take hormones, I’m not female enough, I’m not woman enough, I’m just a man with makeup” […], and she always told me “The hormones, the surgery, these are not things that make you more or less a woman, you are already yourself. Inside, you are already yourself and I can see you”. And this speech saved me.

(L., 24 years)

Another way through which participants felt affirmed consisted in their therapist’s acknowledgment of social oppressions affecting non-binary identities:

At that moment, he did something I needed; that is, he recognized how difficult this path had been and how good I had been in getting this far on my own […] He sees how difficult the [non-binary] condition is and how it is socially oppressed.

(F., 28 years)

Microaffirmations mainly consisted in verbal and non-verbal acts that gave value to participants’ gender and coming out:

I shared with my therapist about the struggle in using public restrooms, and then when I came back the following time, I saw that there were “gender-free toilet” signals attached to the toilet door of the office. And I also discovered that she had a meeting with other colleagues to discuss it and put it in the other studios.

(O., 33 years)

Another key topic of microaffirmations concerns the correct use of names and pronouns. Participants were positively surprised when their therapists asked about their names and pronouns without them having to express such requests:

At one point, she asked me, “Ok, so what pronouns do you want me to use”? To me, this thing was like… like I thought “Ah wow, you know I’m wondering about this thing”? And I said, “Masculine pronoun”, and then she asked me about my name. And from that moment, she used my correct name and pronoun.

(P., 23 years)

#### 3.2.2. Non-Binary Negative Approach through Gender Identity Change Efforts, Manifest Aggressions, and Microaggressions

Negative practices ranged on a continuum from the open rejection of non-binary gender identity to more subtle forms of microaggressions. Some participants reported severe negative interventions acted by their therapist, such as more or less explicit attempts to change their gender identity through invalidation, pathologization, and a conscious refusal to utilize the correct name and pronouns:

Maybe he didn’t have the training to deal with it… Perhaps he didn’t care, or he really didn’t understand. He opposed my gender identity because he invalidated me every time… insistently. He corrected me for using the feminine pronoun each time I used the masculine.

(M., 31 years)

Interviewer: Did your therapist ever want to change your gender identity?

Participant: But emm, since when I started therapy, I was questioning, it happened that I told her, “You know, in certain moments, I feel like I wanted to be a man”, and she answered me, “But what are you saying, you are not a man!” And since I also went there for some dissociation issue, I felt that my therapist thought my doubt about gender identity was due to a form of dissociation.

(B., 29 years)

Other cases were characterized by a manifest aggressive behavior of the therapist as a response to the participant’s attempt to talk about their gender identity:

I tried to say that I felt this gender ambivalence, I asked her if she had ever heard about non-binary and genderqueer identities, but she immediately reacted and criticized me. She thought I wanted to talk about politics and the current debate about gender identity, a construct she is very critical about. Actually, I just wanted to talk about myself. So, the following time I was much more introverted, a little uncomfortable, right? And she told me that I was regressing in therapy as if I had made progress up to that point, and then sharing this experience was, in her opinion, a sign of regression. When I raise these issues, she often says that this is connected to my problems, in that I am hypersensitive to the subject and very reactive to the question, that this is my stuff, a negative transference.

(S., 24 years)

The most common negative interventions reported by participants were microaggressions. In many cases, microaggressions occurred within a positive therapeutic relationship. For that reason, and given the subtle nature of microaggressions, sometimes participants struggled to recognize them as negative practices. For instance, some participants tended to interpret as positive or natural the absence of reaction from their therapist:

Interviewer: How did your therapist react when you came out as non-binary?

Participant: As if nothing happened. I think that for her, it is a normal thing that does not require to be investigated as if I told her my date of birth. She doesn’t care. There has not been a change, like “let’s talk more about this”.

(T., 35 years)

Others noticed that their therapist avoided talking about gender-related issues:

It’s weird because at the beginning, I remember that when […] I referred to gender dysphoria, as well as to my relationship with my sexual orientation, or gender identity, and my relationship with my sexual orientation, or gender identity, it was a bit like if she did… not properly ignoring it, but she didn’t say like “Ah okay, fine, let’s talk about this”. She instead said something like, “ah let’s talk about your family”.

(N., 30 years)

Another example of microaggressions occurred when the therapist invalidated the gender identity experience of participants using stereotypical images associated with non-binarism:

The problem is that people think that [gender] fluidity is an absence of definition or that it depends on a trend of the moment. And this thing, of course, when it is said by your therapist—even in a good way, talking about many other things—is sad, and you don’t always have the strength to say, “No, man, that’s not how it works”.

(F., 33 years)

Finally, a common microaggression consisted of the therapist’s difficulty in using the correct pronouns. The incorrect use may manifest through misgendering, as well as through awkwardness when using the correct pronouns:

I don’t like that every time that my therapist uses feminine pronouns, it creates ridiculous or embarrassing situations. It seems that she just can’t use it, like if it was an effort or if she was reluctant.

(B., 22 years)

### 3.3. The Therapeutic Relationship

#### 3.3.1. Therapeutic Alliance, Disclosure, and Intimacy

A good alliance emerged when participants reported positive feelings associated with the therapeutic relationship. The setting was described as comfortable, and the therapist was perceived as engaged and supportive:

I see that my therapist believes in what she does, and that makes me trust her. She seems to believe in it that people may be non-binary. I think she really understands all my needs.

(O., 33 years)

In this scenario, participants felt comfortable coming out, and the therapist was able to understand and accompany the awareness process:

I noticed that when I spoke to her, she made research by herself [on non-binary issues] to have a dialogue with me in the right way, to be understanding, and I was pleased. Then […] she was able to talk about it better, and she accompanied me towards a better understanding of myself.

(F., 23 years)

#### 3.3.2. Rupture of the Therapeutic Alliance, Concealment, and Dropout

The rupture of the alliance was characterized by a negative description of the therapeutic atmosphere. The climate of the setting was uncomfortable, so participants omitted important information up to the point of concealing their gender identity completely:

No, I don’t feel comfortable talking about my intimate life. Because I’m afraid she can’t understand or that she can be judgmental. Because she often makes nasty comments. No, I don’t feel comfortable sharing certain things.

(S., 24 years)

When the therapist was not able to understand and repair the ongoing rupture, participants often abandoned the therapeutic path:

I felt hostile with her at times, even a little angry […] After my coming out, I was very suffering, and it seemed to me that she didn’t deepen certain things so, after a while, it seemed to me that there was no longer a space for dialogue and so, at a certain point, I decided to stop.

(A., 31 years)

#### 3.3.3. Repairing Alliance Rupture

The reparation of alliance rupture occurs when the therapist tries to restore a positive balance following misunderstandings, disappointments, or breakups. Essential was the ability of the therapist to recognize microaggressions and apologize:

Once she said something that it was better if she didn’t say. Since I was wondering about the possibility of going to XXX [gender-affirming clinic’s name], and she told me, “I don’t recommend it because you look too feminine”. […] Then she apologized, and she explained that she did it to protect me as she knew the type of narrative they require in that clinic. She was afraid that they would tell me things that would hurt me.

(R., 30 years)

The person experiences reparations as events that resolve ruptures and improve trust and the therapeutic alliance.

## 4. Discussion

In recent years, an increasing number of people self-identify as non-binary [12,25,26]. Non-binary subjectivities deal with different issues, such as the need to explore and affirm their identities, struggling with the coming out process, and, as part of a minority group, being affected by minority stress [40,41]. These experiences might lead non-binary people to contact a psychotherapist with the aim of finding support in dealing with their issues. However, non-binary people often face challenges in accessing quality healthcare to live according to their gender worldwide [42] since mental health professionals are not prepared to receive and address the unique needs of this population [43]. 

The professionals’ lack of competence on this topic significantly impacts the non-binary clients’ perception of psychotherapy, and they may experience negative feelings, therapeutic dropout, and avoidance of mental health services [44]. Given the lack of research on this topic, the present study involved non-binary people currently attending or having recently attended psychotherapy. Through Codebook Thematic Analysis [37], we identified three main thematic areas describing non-binary clients’ psychotherapy experiences. The thematic areas were interconnected, extending from (1) the self to (2) the practices of the therapist and how these variables impact (3) the therapeutic relationship. 

The therapist’s self is concerned with how personal and professional aspects are actively and purposefully used to promote the aims of therapy [45]. To “use” the “self” competently and appropriately, therapists must be sufficiently aware of their aspects and prepared for those issues that are meaningful for their clients. In this study, we referred to the therapist’s self by considering the therapist’s sexual identity and their level of knowledge of non-binary issues. When the therapists are aware of some key characteristics related to their sexual identity, they are likely to appear (and are) more competent on non-binary issues. For instance, if the therapist acknowledges their privilege as a cis heterosexual woman or man, it is more likely that a trans/non-binary client will feel more comfortable and safer in the interaction.

Findings from this study showed that non-binary people often prefer to know the therapist’s sexual orientation and gender identity. Some participants reported the desire to engage a non-binary and/or queer therapist to increase the likelihood of feeling understood and minimize the risk of stigma within the clinical setting [46,47]. Since it is tough to find a non-binary therapist in Italy, most participants looked for a cis woman therapist instead, who was perceived as more able to empathize and create a safer environment than a cis man. Such findings can be contextualized and interpreted as a consequence of the Italian sexist culture that promotes cis men’s performance of hegemonic masculinity in most common social interactions. As a result, non-binary people (as well as other marginalized gender identities) may perceive the interactions with cis men as more threatening. 

On the other hand, having a non-binary therapist was reported by some participants as an additional challenge in defining boundaries, especially when they shared the same queer community [48]. Given that queer communities are relatively small in Italy, it is likely that non-binary therapist may encounter their clients at public events and/or face the dilemma of overlapping relationships [49]. Non-binary and queer therapists should be aware and explicit such risks to their clients at the beginning of the therapeutic path to create a clear and comfortable setting.

Most therapists were described as not trained and/or unprepared for non-binary issues. As found in previous research involving transgender clients, participants in this study reconducted the negative experiences with psychotherapy to their therapists’ lack of preparation [47,50]. In some cases, the lack of knowledge was compensated for by open-mindedness and a predisposition to learn, making participants feel respected. Participants proudly described how their therapist improved their knowledge thanks to the encounter with them, perceiving this as an element of the unicity of their relationship. On the contrary, when the lack of knowledge was not accompanied by motivation and empathy, there was a higher risk of experiencing discomfort and breaking away from the setting. Finally, in line with previous studies with transgender clients [51,52], when the therapist was trained, participants reported positive feelings such as esteem and pride for their therapist’s engagement in non-binary issues, and the therapeutic alliance resulted in reinforced [46].

The therapist’s practice consisted of verbal and non-verbal communication that influenced the participants’ experience of the setting. We categorized the therapist’s practice into non-binary affirmative or negative approaches [44]. Affirmative practices occurred when the therapist validated the client’s gender identity through different acts. For instance, participants felt affirmed when their therapist overtly declared seeing their authentic gender and addressed the psychological implications of cisnormativity [23]. Other affirmative acts consisted of microaffirmations and supporting interventions such as the correct use of participants’ names and the use of gender-free toilet signals on the doors of the studio’s bathrooms [53]. Affirmative approaches may involve the acknowledgment of cisgenderism and its effect on non-binary clients’ mental health outcomes; being reflexive about and declaring one’s own gender identity; cultivating knowledge on non-binary issues, medical and social gender-affirming paths; being informed about local resources (e.g., affirmative centers and providers; social/political groups) for trans and non-binary individuals; using correct names and pronouns [2].

The negative approach manifested when the psychotherapist reproduced, both consciously and unconsciously, cisnormative attitudes toward the client. Specifically, cisnormative attitudes and beliefs manifested when the therapist assumed that the client was cisgender, leaving little room for coming out; or when, despite the client’s coming out, the therapist did not respect their chosen name and pronouns and/or referred to the client’s gender identity as something that requires to be treated. Examples of negative and cisnormative approaches consisted of explicit attacks on participants’ gender identity, a pathologizing attitude toward non-binarism, and microaggressions. The most damaging practice was characterized by the therapist’s attempt to change the client’s gender identity by responding to cisnormative pressures [54]. Gender identity change efforts were made through explicit conversion therapies and the therapist’s refusal to recognize the participant’s gender with overt verbal disapproval and intentional misuse of pronouns. Several studies have documented the psychological and social harm associated with gender identity change efforts since they push individuals to conform to expectations that are not congruent with their identity and severely damage their sense of self [43].

Additional negative practices included the therapists’ aggressive response when participants came out as non-binary or tried to discuss gender identity issues. Manifest aggressions were characterized by rigid and manipulative attitudes, pathologizing interpretations of non-binary identities, and verbal aggressions. More subtle forms of negative approach were microaggressions that mainly occurred when the therapist: overemphasized or minimized the role of the client’s gender identity; avoided talking about themes related to gender identity, such as life challenges and coming out; adopted a cisnormative perspective on the client’s experiences and relationships; stereotypically considered non-binarism as a temporary phase, a trend, or confusion towards one’s gender identity; assumed that a non-binary gender identity necessarily leads to negative feelings and needs to be psychologically treated [19,30,55,56]. Non-verbal microaggressions emerged through rigid posture, embarrassment, reluctance, silence, and absent or infantilizing reactions when the client brought issues related to their gender identity in therapy. Verbal microaggressions were typically characterized by adopting a gendered language, misgendering, and deadnaming.

The quality of the interaction significantly impacted the therapeutic alliance. As found in previous studies, when the therapist was able to affirm the non-binary client identity, the therapeutic connection increased, whereas when they adopted a negative approach, the alliance weakened [57]. A good alliance was described through feelings of confidence, collaboration, and intimacy. Participants were completely open about gender identity issues and experiences in this scenario. On the contrary, when the alliance was missed, participants reported discomfort, disappointments, and negative expectations that hindered coming out and led to omitting essential aspects of life, often resulting in therapeutic dropout [19,54]. The experience of shame for one’s gender identity is common among non-binary people as a result of repeated exposure to social rejection of gender non-conformity [58]. For that reason, the therapeutic setting may represent a healing experience when aspects related to gender identity and cisnormativity are addressed by the therapist. On the contrary, when the therapist denies or expresses awkwardness toward the client’s gender identity (e.g., by refusing or showing difficulties in using the correct name and pronouns), the setting reinforces the negative feelings related to shame and pushes the client’s self-concealment.

Finally, episodes of alliance rupture characterized both positive and negative therapeutic relationships. Indeed, ruptures and mistakes occur regardless of a therapist’s attitude and intention [53]. What makes the difference is the overall quality of the therapeutic relationship and how the therapist faces the situation. The alliance rupture based on the client’s gender minority identity may represent a little-known aspect for psychotherapists so that they might be unaware of their therapeutic mistake. Our findings showed that the alliance was most easily repaired when the therapist, rather than the client, addressed the rupture and apologized; that is when the therapist was able to acknowledge and declare their gender identity bias with the client [30].

Looking at the frequency of each sub-theme (Table 1), sexual identity and the level of knowledge of the psychotherapist on non-binary issues seem to represent important topics as they emerged from most of the participants’ narratives [46,47]. Affirmative approaches were reported less frequently than negative approaches. Specifically, microaggressions were the most frequent episodes occurring within the therapeutic setting, thus confirming a gradual shift toward more subtle and unintentional forms of discrimination [19]. Accordingly, alliance ruptures were the most frequent sub-theme of the therapeutic relationship, with only a small percentage of examples of reparations.

### 4.1. Research and Clinical Implications

The findings of the present research have important implications for clinical practice. Thanks to participants’ narratives, we identified those factors that are part of a negative or, instead, an affirmative practice with non-binary clients. We noticed that the therapist’s self, in terms of their sexual identity and professional competence, has an impact on the therapeutic process, even before the process starts [59]. Non-binary people would prefer psychotherapists who are already knowledgeable or have had experiences with non-binary gender-related issues. Moreover, the therapeutic alliance may be influenced—both in a positive and a negative direction—by the therapist’s gender identity [60]. Generally, those therapists who are actively engaged in trans and non-binary communities are perceived as more competent and supportive, thus enhancing positive feelings toward the therapeutic relationship [44,46].

We also found that clinical mistakes, especially microaggressions, are common, even because they can be unintentional and unconscious, thus resulting also in positive therapeutic relationships. Given the cisgenderist norms that prevail in our societies, it is almost inevitable that therapists internalize and make clinical errors that hurt the client. Moreover, some good practices, such as using chosen names and pronouns correctly, are relatively new and unfamiliar to practitioners approaching clinical work with non-binary clients. However, microaggressions may lead to alliance rupture and therapeutic dropout, especially if the therapist does not efficiently address them.

Mental health professionals should incorporate issues related to gender minority identities in their clinical training to provide a welcoming and comfortable setting to non-binary people who require their support. Moreover, it is fundamental to recognize and deconstruct the internalized gender biases and, when clinical errors occur, acknowledge and use them to increase connection with their client. Finally, to embrace an affirmative approach with non-binary clients, it is also necessary to adopt active listening, open-mindedness, and an overall predisposition to learn through the client’s experience giving value to their precious contribution.

### 4.2. Limitations and Future Research

The present study is not exempt from limitations which must be considered for the generalizability of the findings to the entire non-binary population. First, all participants were White and Italian; thus, we were not able to consider the role of ethnicity as a potential additional factor influencing the experience of psychotherapy. We acknowledge that racialized people are underrepresented in psychological research and invisibilized in the public discourse on sexual and gender minorities. Future research should address this gap, giving voice to those subjectivities incorporating multiple oppressions and identifying their unique experiences of psychological settings.

Moreover, qualitative research is increasingly recognized as fundamental to addressing life experiences’ complexity. However, qualitative methods require a great amount of energy for a relatively small group of participants, limiting the generalizability of the results. The present study could inspire the construction of quantitative measures that might be distributed to a larger sample of participants.

Finally, we focused on participants’ narratives of psychotherapy, that is, their perception of the setting and the therapist’s attitudes. Consequently, we cannot assume the objectivity of information emerging from the interviews, as they might be influenced by participants’ feelings associated with transfer and projections. Nevertheless, the client’s feelings about the clinical setting have a concrete impact on the therapeutic alliance and adherence to the psychotherapy. We invite future studies to assess the therapists’ experience of their work with non-binary clients to expand knowledge on the therapists’ attitudes, awareness, competence, and sense of efficacy when dealing with non-binary clients.

## 5. Conclusions

There is evidence that sexual and gender minority people present worse mental health conditions if compared to the general population [4,61]. There is a growing need to identify and differentiate the specific forms of stigma affecting the different subgroups of the LGBTQIA+ population in order to increase knowledge and improve the quality of psychological support in clinical settings [62,63]. The research on non-binary people is still lacking, and there is a need to conduct additional studies able to identify the unique forms of stigma and resilience associated with such a population.

Despite the limitations, the present study provides interesting information for the clinical practice with non-binary clients. In recent years, many people have identified beyond the gender binary, especially among youth [12,26], and non-binary people and their families may require psychological support to understand and manage the issues and challenges resulting from a non-binary identity [41]. However, this study’s findings showed that psychotherapists are often unaware of and unprepared to deal with non-binary gender identities because of the absence of research and guidance on this topic. Consequently, they may commit clinical mistakes whose seriousness strictly depends on the overall quality of the therapeutic alliance [64]. 

Episodes of aggression and microaggression in clinical settings increase the stigma-related effects on mental health [19,30]. On the contrary, affirmative experiences in psychotherapy may significantly enhance well-being and empowerment, offering useful instruments to counterbalance the detrimental impact of societal cisgenderism [23]. As mental health professionals and researchers, it is fundamental to increase knowledge of those practices that promote non-binary clients’ well-being, such as the correct use of names and pronouns, the validation of their gender identity, and the recognition of the impact of cisgenderism on well-being [53].

## Figures and Tables

**Table 1:** Thematic structure, representative quotations, and frequencies.

Theme	Sub-Theme	Frequency	Quotation
**The self of the psychotherapist**	Gender identity and sexual orientation	76%	According to me, it is important to know the gender identity or sexuality of a therapist […] as it creates a “safe space” and makes me feel that there is a basic understanding that I could never find in a cis or straight person. (B., 22 years)
	Knowledge and experiences	84%	I think she didn’t know what the binder was because I was talking about that, and I realized she wasn’t following me, and she didn’t even tell me. This made me a little uncomfortable because I didn’t want to explain, and it also bothered me that she didn’t tell me that she wasn’t following me. (K., 24 years)
**The practice of the psychotherapist**	Non-binary affirmative approach		
	Validation	32%	She often tells me “Your trans path has already begun, you are another person, and you have made an incredible internal and external transformation”. When she looks at me, it is as if I can see my identity, what I am inside. (M., 33 years)
	Microaffirmations	28%	She is supportive; she immediately asked me about pronouns and used them as the most natural thing. (D., 28 years)
	Non-binary negative approach		
	Gender identity change efforts	12%	I went to this psychologist that received people who needed support in the university chapel. She told me she also “helped” homosexual guys who wanted to change their sexual orientation. And then, when I went there at times, she said to me that I did not respect the normal development, that identification occurs typically with the parent of one’s sex, and therefore I apparently was deviant. (S., 24 years)
	Manifest aggressions	32%	Towards the end of the first session, she says, “Yes but now I have to ask you this thing about the name because I can’t get over it”. And she started saying that my name didn’t make sense and that it didn’t exist, that I should have changed it. (M., 26 years)
	Microaggressions	72%	During the first session, she asked me to fill out a personal data sheet where there were only two options for gender, male or female, and then I said “Eh sorry, I am neither of them” and she said to me “Oh yes they are a bit outdated”, and then I said “Well, change them” and she told me “Ok, but in the meantime put one at random and then we’ll sign a note”. (L., 25 years)
**The therapeutic relationship**	Therapeutic alliance	40%	I feel like it has been a typical growth [with the therapist] which, however, started from a very good basis, from a profound understanding of the whole social aspect, of the relationship between society and gender non-conformity. (F., 28 years)
	Therapeutic alliance rupture	68%	I had the feeling that for her non-binary identities were linked to themes such as indecision and confusion. Sometimes, I wasn’t taken seriously. It was hard. I had intrusive thoughts about therapy. I mean, after two years, I should feel comfortable in front of you. (B., 22 years)
	Repairing alliance rupture	20%	Often my therapist, at first, was wrong to say my name. Also because it is a strange name, let’s say. And I didn’t say anything because I didn’t want to appear exaggerated. Then he realized and said: “This is an important thing that deserves attention!”. (B., 30 years)

## Data Availability

Anonymized data will be made available upon reasonable request to the corresponding author.

## References

[1] American Psychological Association (2015). Guidelines for psychological practice with transgender and gender nonconforming people. Am. Psychol..

[2] Barker M.J., Iantaffi A., Richards C., Bouman W.P., Barker M.J. (2017). Psychotherapy. Genderqueer and Non-Binary Genders.

[3] Ansara Y.G., Berger I. (2016). Cisgenderism. The Wiley Blackwell Encyclopedia of Gender and Sexuality Studies.

[4] Tan K.K., Treharne G.J., Ellis S.J., Schmidt J.M., Veale J.F. (2019). Gender minority stress: A critical review. J. Homosex..

[5] Jones B.A., Pierre Bouman W., Haycraft E., Arcelus J. (2019). Mental health and quality of life in non-binary transgender adults: A case control study. Int. J. Transgend..

[6] Rimes K.A., Goodship N., Ussher G., Baker D., West E. (2019). Non-binary and binary transgender youth: Comparison of mental health, self-harm, suicidality, substance use and victimization experiences. Int. J. Transgend..

[7] Cheung A.S., Leemaqz S.Y., Wong J.W., Chew D., Ooi O., Cundill P., Silberstein N., Locke P., Zwickl S., Grayson R. (2020). Non-binary and binary gender identity in Australian trans and gender diverse individuals. Arch. Sex. Behav..

[8] Jellestad L., Jäggi T., Corbisiero S., Schaefer D.J., Jenewein J., Schneeberger A., Kuhn A., Garcia Nuñez D. (2018). Quality of life in transitioned trans persons: A retrospective cross-sectional cohort study. Biomed Res. Int..

[9] Thorne N., Witcomb G.L., Nieder T., Nixon E., Yip A., Arcelus J. (2019). A comparison of mental health symptomatology and levels of social support in young treatment seeking transgender individuals who identify as binary and non-binary. Int. J. Transgend..

[10] Scandurra C., Mezza F., Maldonato N.M., Bottone M., Bochicchio V., Valerio P., Vitelli R. (2019). Health of non-binary and genderqueer people: A systematic review. Front. Psychol..

[11] Burgwal A., Gvianishvili N., Hård V., Kata J., García Nieto I., Orre C., Smiley A., Vidić J., Motmans J. (2019). Health disparities between binary and non binary trans people: A community-driven survey. Int. J. Transgend..

[12] Todd K., Peitzmeier S.M., Kattari S.K., Miller-Perusse M., Sharma A., Stephenson R. (2019). Demographic and behavioral profiles of nonbinary and binary transgender youth. Transgend. Health.

[13] Hagen D.B., Galupo M.P. (2014). Trans* individuals’ experiences of gendered language with health care providers: Recommendations for practitioners. Int. J. Transgend..

[14] Lykens J.E., LeBlanc A.J., Bockting W.O. (2018). Healthcare experiences among young adults who identify as genderqueer or nonbinary. LGBT Health.

[15] Ansara Y.G., Hegarty P. (2014). Methodologies of misgendering: Recommendations for reducing cisgenderism in psychological research. Fem. Psychol..

[16] Baldwin A., Dodge B., Schick V.R., Light B., Schnarrs P.W., Herbenick D., Fortenberry J.D. (2018). Transgender and genderqueer individuals’ experiences with health care providers: What’s working, what’s not, and where do we go from here?. J. Health Care Poor Underserved.

[17] Baril A., Trevenen K. (2014). Exploring ableism and cisnormativity in the conceptualization of identity and sexuality ‘disorders’. Ann. Rev. Crit. Psychol..

[18] Veale J.F., Byrne J.L., Tan K.K.H., Guy S., Yee A., Nopera T., Bentham R. (2019). Counting Ourselves: The Health and Wellbeing of Trans and Non-Binary People in Aotearoa New Zealand.

[19] Morris E.R., Lindley L., Galupo M.P. (2020). “Better issues to focus on”: Transgender Microaggressions as Ethical Violations in Therapy. Couns. Psychol..

[20] Sue D.W., Capodilupo C.M., Sue D.W., Sue D. (2007). Racial, gender and sexual orientation microaggressions: Implications for counseling and psychotherapy. Counseling the Culturally Diverse: Theory and Practice.

[21] Shelton K., Delgado-Romero E.A. (2011). Sexual orientation microaggressions: The experience of lesbian, gay, bisexual, and queer clients in psychotherapy. J. Couns. Psychol..

[22] Pulice-Farrow L., Bravo A., Galupo M.P. (2019). “Your gender is valid”: Microaffirmations in the romantic relationships of transgender individuals. J. LGBT Issues Couns..

[23] Anzani A., Morris E.R., Galupo M.P. (2019). From absence of microaggressions to seeing authentic gender: Transgender clients’ experiences with microaffirmations in therapy. J. LGBT Issues Couns..

[24] O’Shaughnessy T., Speir Z. (2018). The state of LGBQ affirmative therapy clinical research: A mixed-methods systematic synthesis. Psychol. Sex. Orientat. Gend. Divers..

[25] Mirabella M., Piras I., Fortunato A., Fisher A.D., Lingiardi V., Mosconi M., Ristori J., Speranza A.M., Giovanardi G. (2022). Gender Identity and Non-Binary Presentations in Adolescents Attending Two Specialized Services in Italy. J. Sex. Med..

[26] Scandurra C., Carbone A., Baiocco R., Mezzalira S., Maldonato N.M., Bochicchio V. (2021). Gender Identity Milestones, Minority Stress and Mental Health in Three Generational Cohorts of Italian Binary and Nonbinary Transgender People. Int. J. Environ. Res. Public Health.

[27] Baiocco R., Pistella J. (2019). “Be as you are” clinical research center at the Sapienza University of Rome. J. Gay Lesbian Ment. Health.

[28] Gheno V. (2020). Ode to the Schwa. Lo Schwa tra Fantasia e Norma. Come Superare il Maschile Sovraesteso Nella Lingua Italiana. Lafalla. https://lafalla.cassero.it/lo-schwa-tra-fantasia-e-norma/.

[29] Gheno V. (2022). Questione di privilegi: Come il linguaggio ampio può contribuire ad ampliare gli orizzonti mentali. [A matter of privileges: How an inclusive, or broad, language can help broaden mental horizons]. AG About Gender.

[30] Johnson D.E. (2014). The Impact of Microaggressions in Therapy on Transgender and Gender-Nonconforming Clients: A Concurrent nested Design Study. Ph.D. Thesis.

[31] Lingiardi V., Capozzi P. (2004). Psychoanalytic attitudes towards homosexuality: An empirical research. Int. J. Psychoanal..

[32] Twist J., de Graaf N.M. (2018). Gender diversity and non-binary presentations in young people attending the United Kingdom’s National Gender Identity Development Service. Clin. Child Psychol..

[33] Titman N. How Many People in the United Kingdom are Nonbinary?. 2014..

[34] Garrison S. (2018). On the limits of “trans enough”: Authenticating trans identity narratives. Gend. Soc..

[35] Nicolazzo Z. (2016). ‘It’s a hard line to walk’: Black non-binary trans* collegians’ perspectives on passing, realness, and trans*-normativity. Int. J. Qual. Stud..

[36] Galupo M.P., Pulice-Farrow L., Pehl E. (2021). “There Is Nothing to Do About It”: Nonbinary Individuals’ Experience of Gender Dysphoria. Transgend. Health.

[37] King N., Symon G., Cassell C. (2012). Doing template analysis. Qualitative Organization Research: Core Methods and Current Challenges.

[38] Braun V., Clarke V. (2022). Conceptual and design thinking for thematic analysis. Qual. Psychol..

[39] Braun V., Clarke V. (2021). One size fits all? What counts as quality practice in (reflexive) thematic analysis?. Qual. Res. Psychol..

[40] Lefevor G.T., Boyd-Rogers C.C., Sprague B.M., Janis R.A. (2019). Health disparities between genderqueer, transgender, and cisgender individuals: An extension of minority stress theory. J. Couns. Psychol..

[41] Scandurra C., Vitelli R., Maldonato N.M., Valerio P., Bochicchio V. (2019). A qualitative study on minority stress subjectively experienced by transgender and gender nonconforming people in Italy. Sexologies.

[42] Jarrett B., Peitzmeier S.M., Restar A., Adamson T., Howell S., Baral S., Beckham S.W. (2020). Gender-affirming care, mental health, and economic stability in the time of COVID-19: A global cross-sectional study of transgender and non-binary people. medrXiv.

[43] American Psychological Association (2021). APA Resolution on Gender Identity Change Efforts.

[44] McCullough R., Dispenza F., Parker L.K., Viehl C.J., Chang C.Y., Murphy T.M. (2017). The counseling experiences of transgender and gender nonconforming clients. J. Couns. Dev..

[45] Horne K.B. (1999). The relationship of the self of the therapist to therapy process and outcome: Are some questions better left unanswered?. Contemp. Fam. Ther..

[46] Benson K.E. (2013). Seeking support: Transgender client experiences with mental health services. J. Fem. Fam. Ther..

[47] Rachlin K. (2002). Transgender individuals’ experiences of psychotherapy. Int. J. Transgend..

[48] Schank J.A., Helbok C.M., Haldeman D.C., Gallardo M.E. (2010). Challenges and benefits of ethical small-community practice. Prof. Psychol. Res. Pract..

[49] Heins N. (2012). Queer Predicaments: Dual Relationships in Sexual Minority and Gender Variant Clinicians Who Practice within Their Communities. Ph.D. Thesis.

[50] Fortunato A., Giovanardi G., Mirabella M., Di Ceglie D., Speranza A.M., Caviglia G., Lingiardi V. (2020). Caring for gender diverse children and adolescents in Italy: A mixed-method investigation of clinicians’ knowledge and approach to clinical practice. Clin. Child Psychol. Psychiatry.

[51] Budge S.L. (2013). Interpersonal psychotherapy with transgender clients. Psychotherapy.

[52] Giovanardi G., Mundo E., Lingiardi V. (2021). Paola on the couch: The quest for feminine identity in an empirically supported psychoanalytic psychotherapy of a trans woman. Psychoanal. Psychol..

[53] Knutson D., Koch J.M., Goldbach C. (2019). Recommended terminology, pronouns, and documentation for work with transgender and non-binary populations. Pract. Innov..

[54] James S.E., Herman J.L., Rankin S., Keisling M., Mottet L., Ana M. (2016). The Report of the 2015 U.S. Transgender Survey. National Center for Transgender Equality. https://transequality.org/sites/default/files/docs/usts/USTS-Full-Report-Dec17.pdf.

[55] Elder A.B. (2016). Experiences of older transgender and gender nonconforming adults in psychotherapy: A qualitative study. Psychol. Sex. Orientat. Gend. Divers..

[56] Hunt J. (2014). An initial study of transgender people’s experiences of seeking and receiving counselling or psychotherapy in the UK. Couns. Psychother. Res..

[57] Safran J.D., Muran J.C., Eubanks-Carter C. (2011). Repairing alliance ruptures. Psychotherapy.

[58] Carvalho S.A., Carvalho F., Fonseca L., Santos G., Castilho P. (2022). Beyond the centrality of shame: How self-concealment and fear of receiving compassion from others impact psychological suffering in transgender adults. J. Homosex..

[59] Rosati F., Pistella J., Baiocco R. (2021). Italian sexual minority older adults in healthcare services: Identities, discriminations, and competencies. Sex. Res. Soc..

[60] Kahn J., Dodd S.J. (2021). Therapist Self-Disclosure: Use of Self as a Transgender Therapist. The Routledge International Handbook of Social Work and Sexualities.

[61] Meyer I.H. (2003). Prejudice, social stress, and mental health in lesbian, gay, and bisexual populations: Conceptual issues and research evidence. Psychol. Bull..

[62] Rosati F., Coletta V., Pistella J., Scandurra C., Laghi F., Baiocco R. (2021). Experiences of Life and Intersectionality of Transgender Refugees Living in Italy: A Qualitative Approach. Int. J. Environ. Res. Public Health.

[63] Rosati F., Pistella J., Ioverno S., Baiocco R. (2018). Relational variables and psychological well-being in lesbian, gay, bisexual and transgender elders: A critical review. G. Ital. Psicol..

[64] Mizock L., Lundquist C. (2016). Missteps in psychotherapy with transgender clients: Promoting gender sensitivity in counseling and psychological practice. Psychol. Sex. Orientat. Gend. Divers..

